# Concerning Value of Partial Operations in the Treatment of Cancer of the Cervix

**Published:** 1892-04

**Authors:** E. Monod

**Affiliations:** Bordeaux, France


### *TRANSLATION.*

### CONCERNING THE VALUE OF PARTIAL OPERATIONS IN THE TREATMENT OF CANCER OF THE CERVIX.

BY DR. E. MONOD, BORDEAUX, FRANCE.

[Translated from the *Archives de Tocologie et de Gynecologie*, by DR. L. B. GRANDY, Atlanta, Ga.]

Since the memorable discussions which it aroused in the Society of Surgery in the year 1888, the question of the surgical treatment of cancer of the uterus seems to have been the order of the day. The medical journals and the transactions of learned societies are filled with facts which relate to it. If one were to judge by the abundance of published papers it would seem that this important question of therapeutics had taken a very decided step in advance, and had even been almost definitely settled. But this is not so, and we do not hesitate to say that the practitioner, as soon as he has made the sad diagnosis of cancer of the womb, finds himself very often greatly embarrassed in determining his line of conduct.

Two rival methods are to-day in practice:

*Partial hysterectomy;* or amputation of the cervix.

*Total hysterectomy.*

Since Czerny, Billroth and Schrœder, in Germany, and Péan and Demons, in France, have reinstated the operation of Récamier under the antiseptic methods, total removal of the uterus, so far as its gravity is concerned, can sustain a comparison with the great operations which are boldly made for cancer in other parts of the body. The mortality, according to the most recent statistics, does not exceed ten to fifteen per cent.

The gravity of the operation, then, not being an obstacle, why is it that we do not apply to uterine cancer the same rules accepted for external cancer in general, which require extirpation as soon as the malignant nature of the tumor has been diagnosticated? There is not a surgeon to-day who, in the presence of even a very circumscribed tumor of the breast, would hesitate to sacrifice the entire gland, be its nature ever so little suspected.*

Why should this surgical principle, so well recognized when applied to the mamma, cease when the uterus is the seat of the lesion? Logic, it seems to me, demands the complete extirpation of an organ, the seat of an epithelial ulceration limited to the cervix, with the same rigor which indicates the removal of the entire breast for a simple cancerous nodule.

Unfortunately, in the results first published, only successful operations were announced; but it was not long before some reservations had to be made on the question of therapeutic success. It was recognized that failures were made, numerous and discouraging. Certain surgeons, formerly convinced advocates of the radical operation, relinquished their previous enthusiasm. As a matter of fact, contrary to that which theory would logically permit us to expect, recurrences are not only just as frequent but also just as rapid after operations apparently radical as after those only partial. We are now speaking generally; for we shall finally see that in the actual state of the matter it is impossible to affirm anything precisely concerning the relative therapeutic values of the two methods.

Permit me now to appeal to some figures. Of forty cases of total hysterectomy reported by Schrœder from his practice from 1878 to 1886, seven remained free from the disease at the end of two years; of thirty-one cases, six remained cured at the end of three years; of eighteen cases, none were cured at the end of four years. Hofmeier's results show sixty-three per cent. of recoveries at the end of one year; twenty-four per cent. at the end of two years; and not one at the end of three years. Over against these discouraging results it is only fair to

* Il n'est pas de chirurgien aujourd'hui qui, en presence d'une tumeur meme tres limitee du sein, hesite, pour peu que sa nature soit suspecte, a sacrifier la totatité de la glande.

place the peculiarly happy statistics of Leopold, extending over his practice for a period of five and a half years. Of eighty operated cases, twenty-seven remained without recurrence for more than two years. Of eighteen hysterectomies, Terrier reports four deaths, ten recurrences, one cure (the lesion being probably only chronic metritis), and three cases which lived one year, twenty-one months, and thirty-three months respectively.

In recent discussions the partial operation, as applied to cancer of the uterus, has found an ardent advocate in Professor Verneuil. He reports twenty-two intra-vaginal amputations of the cervix with the linear ecraseur. Of these nine rapidly recurred; two cases lived for seventeen months and seven years respectively, without local reappearance of the lesion and died from other causes; five patients were lost sight of, in very good condition, three years after the operation; in two cases the patients were doing well at the end of seventeen months and five months respectively; twice metastases occurred after three years of apparent cure; one patient died of peritonitis, and in one case the operation had been done only three months previously.

Such figures carry with them a lesson, and should be taken carefully into account in the discussions as to the therapeutic value of operations addressed to cancer of the uterus. They are interesting to consult, but they have only a relative value if one seeks to deduce from them the superiority of one method over the other. In order that such a deduction may be legitimate it is necessary that the facts composing the statistics should be comparable. But one can safely say that a great number of cases which figure above are absolutely dissimilar. This statement will not surprise us much if we consider the various conditions which ought to be satisfied by statistics having any pretension to uniformity. Let us recall some of these multiple elements:

1. *The age of the lesion*, or the time that has elapsed since the beginning of the disease and the date of operation.

2. *The primary seat of the growth.* Cancer of the body, so far as prognosis goes, should be distinctly separated from cancer of the neck.

3. *The extension of the lesion to neighboring parts.* It is useless to insist on the necessity of establishing a distinct group of cases in which the vagina is, or is not, involved, the abdominal ganglia the seat of secondary tumors, or free from them, etc.

4. *The anatomical form of the lesion.* It is important to determine the clinical form of the cancer, whether *papillary*, *nodular* or *ulcerating*.

5. Finally, *the histological variety of the lesion.* There is not *a* cancer of the uterus, but there are *cancers* of the uterus, and there is no doubt that to the different histological varieties should correspond different clinical histories.

Concerning cancer of the cervix it is necessary to recognize two classes of well defined cases:

I. *The disease is already well advanced when the surgeon is consulted.* In this group are classed those numerous cases in which the uterus is immovable in the pelvis, and in which the lesion has involved the vaginal wall. This double state of affairs precludes all attempts at a curative operation, either partial or radical. One finds himself here in the presence of a great class of inoperable cancers. We insist on the great importance which attaches to *involvement of the vagina* as a contraindication to extirpation.

It is to these cases, desperate in appearance, that the palliative operations, such as curetting and partial excision, address themselves; from which patients derive an incontestable benefit, and which assures to them often a survival surpassing their hopes.

[For examples, the author here reports two cases from his practice. One, of epithelioma of the cervix, with wide involvement of the vagina; had existed two years; curetted, survived one year. The other, nodular cancer with extension to vagina; had existed one year; curetted; return to comparative health; survived one year and eight months.—L. B. G.]

II. *The disease has only begun when the surgeon is consulted.* Two classes of these cases are met with: (a) In spite of the small extent of the lesion and its apparent localization, there remains some doubt as to its higher limit. In this category belong the nodular and ulcerating varieties. In one form, as in the other, whether it is a question of circumscribed or infiltrated nodules in the parenchyma of the cervix, or whether there is developed an ulceration on the mucous membrane of the cervical canal, it is almost impossible to say that there do not exist some neoplastic foci further off, or that the erosion has not already attacked the mucous membrane of the body of the uterus. We think that, in these cases, after the diagnosis is well established, and there being no other contraindication, total hysterectomy remains the operation of choice. (b) The lesion is so clearly localized that one is sure of being able to go above its highest limit by simple amputation of the neck. The papillary form (cauliflower-like) represents this class. It is known that this variety of cancer begins as a superficial lesion situated below the vaginal insertion, and that it remains for a long time confined to the surface.

It is in these cases and these alone that the merits of partial and total hysterectomy may be compared.

[Author here recites two cases. One, papillary epithelioma of the cervix; intra-vaginal amputation of the neck with the linear ecraseur; complete recovery two years and a half after the operation. The other, cancer same; five successive excisions; patient doing well seven years and a half after first operation.—L. B. G.]

In order to judge of the relative value of the two methods in the treatment of uterine cancer it will be necessary to group together the facts relating to the class of cases which now occupy us. We do say that, in our mind, the partial method which can be done by every practitioner, mild in its immediate effect and at least encouraging in its ultimate results, ought, in the actual state of the case, to be preferred to operations called *radical*, when these particular cases are observed.

---

THE INTERNATIONAL MEDICAL MAGAZINE.—We have received the first issue of this new medical monthly from the press of the J. B. Lippincott Company. This first number has some able and widely-known contributors, and the matter is of the highest order of scientific merit. The new magazine makes more than a creditable beginning and, we doubt not, will both deserve and attain success.

